# Investigating the benefits of metagenomic next-generation sequencing for patients experiencing infections after total hip replacement surgery: a retrospective cohort study with a minimum of one year of follow-up

**DOI:** 10.3389/fcimb.2026.1735867

**Published:** 2026-02-02

**Authors:** Jiaqing Zhu, Tianwei Xia, Lu Wang, Xindong Yin, Yong Ma, Jirong Shen

**Affiliations:** 1 Department of Orthopedics and Traumatology, Affiliated Hospital of Nanjing University of Chinese Medicine, Nanjing, China; 2 Department of General Surgery, Affiliated Hospital of Nanjing University of Chinese Medicine, Nanjing, China

**Keywords:** bacterial culture, diagnosis, metagenomic next-generation sequencing, periprosthetic infection, total hip arthroplasty

## Abstract

**Objective:**

To explore the clinical significance of metagenomic next-generation sequencing (mNGS) technology in diagnosing and treating periprosthetic joint infection (PJI) following total hip arthroplasty (THA).

**Methods:**

From September 2018 to September 2024, 15 patients with periprosthetic infection after total hip arthroplasty were admitted. There were 11 males and 4 females; ages ranged from 28 to 87 years old, with an average of 63 years old. Infection occurred 6 to 42 months after total hip arthroplasty, with an average of 22.7 months. The infection lasted between 15 and 115 days, averaging 37.6 days. After being admitted to the hospital, joint fluid was collected for bacterial culture and mNGS. Following admission, joint fluid was collected for bacterial culture and mNGS, and antibiotics were adjusted based on the results, with surgery used to control the infection if needed.

**Results:**

Bacterial culture method was positive in 10 cases (66.7%), with a total of 12 pathogenic bacteria types detected. MNGS was positive in 15 cases (100.0%), with a total of 19 pathogenic bacteria types detected. There was a statistically significant difference in the positive rate between the two methods (P < 0.05). Out of the 10 patients, 5 who tested positive using both the bacterial culture method and mNGS test showed identical pathogenic bacterial types, resulting in a 50.0% compliance rate. The testing time (from sample delivery to results) was (3.07 ± 0.96) days for bacterial culture method and (1.67 ± 0.49) days for mNGS test, and the difference was statistically significant (t=5.03, P<0.001). The patients were followed up for 13 to 82 months, with a mean of 40.7 months. In one patient, the infection returned three months after undergoing one-stage revision surgery, while the other 14 patients showed no signs of infection, resulting in an infection control rate of 93.3%.

**Conclusion:**

MNGS can detect the pathogenic bacteria of postoperative PJI after THA more quickly and accurately than the bacterial culture method, which is crucial for guiding antibiotic and surgical treatment combinations for patients with postoperative PJI after THA.

## 1 Introduction

Total hip arthroplasty (THA) is one of the most successful surgical procedures in the 21st century, and is widely used for femoral neck fracture, femoral head necrosis, congenital hip dysplasia, and other diseases. Artificial total hip arthroplasty is used to improve patient function, relieve hip pain, and improve patient quality of life by remodeling the prosthesis to replace the hip joint (Kobayashi et al., 2000; Matheis and Stöggl, 2018; Humphreys and Hoffman, 2020). With the aging of the population worldwide, the number of patients undergoing total hip arthroplasty is increasing year by year, and the number of patients experiencing post-total hip arthroplasty complications is also increasing. There are many factors that limit the service life of the prosthesis, among which periprosthetic joint infection (PJI) is one of the most common causes. It has been found that PJI can complicate 0.39%-3.9% of primary joint replacement surgeries (Yoon et al., 2023). Once PJI occurs, it prolongs the patient’s hospitalization, increases his/her costs, and leads to joint dysfunction.

Bacterial culture is the recognized standard for detecting patients with PJI and has the advantages of low cost and easy detection, but due to its low sensitivity and specificity, bacterial culture is not very significant for the diagnosis of patients with early PJI (Linjie et al., 2021). Metagenomic next-generation sequencing (mNGS) technology is an emerging diagnostic technique for comprehensive analysis of microorganisms and genetic material in samples sent for testing, which can make up for the shortcomings of traditional molecular analyses that are only targeted at detecting a limited number of pathogens with specific primers or probes (Zhang et al., 2019). The emergence of drug-resistant bacteria in PJI has led to the ineffective antibiotic treatment alone. The use of mNGS technology, which detects more rapidly and accurately, to guide the combined application of antibiotics is expected to solve this problem (Zijun et al., 2024).(deleted). From September 2018 to September 2024, we admitted 15 patients with PJI after THA surgery, took bacterial culture and mNGS test to clarify the type of pathogenic bacteria for the patients’ hip synovial fluid, and chose sensitive antibiotics to control the infection in combination with surgery based on the test results, and obtained satisfactory results. The report is as follows.

## 2 Materials and methods

### 2.1 Patient selection criteria

Inclusion criteria: 1. the diagnosis of PJI was in accordance with the diagnostic criteria of the American Musculoskeletal Infection Society in 2013 (Patel et al., 2016); 2. the infected joint fluid was sent for routine bacterial culture and mNGS testing at the same time; 3. the follow-up time was more than 1 year.

Exclusion criteria: 1. mental, language, psychological and other obstacles can not cooperate with the treatment; 2. combined malignant tumors, autoimmune diseases, etc.; 3. patients who died during the follow-up process.

### 2.2 Population study

The study included 15 patients in total, with 11 being male and 4 being female; ages ranged from 28 to 87 years, with an average of 63 years. Type of replacement prosthesis: all were biologically fixed prostheses(Titanium prosthesis, ceramic or polyethylene lining, ceramic femoral head). Infection occurred 6–42 months after replacement surgery, with an average of 22.7 months; the main clinical symptoms were elevated local skin temperature, formation of sinus tracts, and pus flow. The infection lasted between 15 and 115 days, averaging 37.6 days. The preoperative Erythrocyte sedimentation rate (ESR) was 20~88 mm/1 h, with an average of 47.9 mm/1 h. The C-reactive protein (CRP) was 4.7~318.0 mm/L, with an average of 130.1 mg/L. The white blood cell count (WBC) was (3.6~24.6) ×109/L, with an average of 10.1×109/L; The absolute neutrophil count (ANC) was (1.9~67.2) ×109/L, with an average of 12.0×109/L. The general information is shown in **Tables 1** and **2**.

**Table 1:** Clinical data of the patients.

No.	Age	Infection duration (Days)	Whether antibiotics were used before admission	Bacterial culture results (OTUs)	MNGS results(OTUs)
1	28	15	No	1	1
2	75	17	Yes	2	2
3	54	27	No	1	1,4,7
4	68	43	No	–	1,2
5	53	89	Yes	1	1,8
6	75	23	No	–	1
7	64	19	No	1	1,4
8	59	22	No	1	1
9	33	28	No	1	1
10	55	37	No	–	1
11	77	33	Yes	1,3	1,3,9
12	72	41	Yes	2,5	2,5
13	87	29	No	–	4
14	79	26	Yes	–	5
15	62	115	Yes	1,4,6	1,4,6

**Table 2:** Comparison of test data.

No.	ESR (mm/1 h)		CRP (mg/L)		WBC (×109/L)		ANC (×109/L)	
	Preoperative	Postoperative	Preoperative	Postoperative	Preoperative	Postoperative	Preoperative	Postoperative
1	50	41	121.0	7.2	8.7	6.2	6.8	3.8
2	46	33	91.4	16.3	4.8	9.2	67.2	7.5
3	72	56	318.0	16.8	22.4	7.9	20.9	5.7
4	51	35	282.0	8.3	8.7	4.5	5.7	2.6
5	21	18	169.0	44.8	16.4	12.0	8.7	10.4
6	35	29	88.0	3.3	6.2	5.2	4.4	2.7
7	56	43	9.4	79.0	7.3	8.0	4.7	5.5
8	68	51	283.0	22.9	24.6	5.8	22.5	3.9
9	37	26	102.0	11.9	4.2	10.2	2.8	8.6
10	28	23	78.5	27.1	11.5	5.9	9.4	4.2
11	43	28	76.5	45.4	3.6	5.0	1.9	4.4
12	76	49	4.7	5.2	4.7	6.8	2.5	5.6
13	20	15	60.8	12.8	15.0	8.8	13.0	7.6
14	28	23	76.5	68.2	8.7	8.6	6.5	5.8
15	88	52	190.6	9.3	4.4	6.4	2.9	4.2

The two assays were categorized with reference to bacterial operational taxonomic units (OTUs), and there were three categories (nine types) of OTUs: (1) Thick-walled phylum: OTU1 (*Staphylococcus aureus, Staphylococcus epidermidis, Staphylococcus simulans, Staphylococcus simulans, and Streptococcus hemolyticus*), OTU3 (*Enterococcus faecalis*), OTU4 (*Streptococcus mitis, Streptococcus oralis, pyogenic streptococcus, Streptococcus dysgalactiae*); ② Aspergillus phylum: OTU5 (*Pseudomonas aeruginosa*), OTU6 (*Baumannii*), OTU7 (*Enterobacter chuandaensis*); ③ Other groups: OTU2 (*Mycobacterium Houstonese, Mycobacterium tuberculosis*), OTU8 (*Desulfomonilaceae*), OTU9 (*Aspergillus*).

### 2.3 Treatment

#### 2.3.1 Pathogen detection

1. Specimen collection: All patients were punctured preoperatively, and a sterile syringe was used to obtain joint fluid samples from the core site of the infected lesion, taking care to avoid mixing with blood.
2. Bacterial culture: The joint fluid specimens were cultured for microorganisms, and the VITEK2 system (VITEK^®^ 2 Compact, bioMérieux; China) was used to identify the strains of bacteria in all the samples and to determine the drug sensitivity.
3. mNGS assay: Nucleic acid high-throughput sequencing was performed by Hangzhou Jieyi Biotechnology Co., Ltd (11 cases) or Guangzhou Weiyuan Genetic Science and Technology Co., Ltd (4 cases) on the joint fluid specimens and analyzed by comparing with microbial-specific databases (Gu et al., 2019), and then special algorithms were used to find out the species of the target microorganisms to obtain the reporting parameters (Miao et al., 2018). Sequencing was performed on the Illumina platform, which includes the following three steps: ① library preparation: fragmentation of the sample DNA and addition of specific junctions to the two segments to construct the sequencing library. ② Sequencing: the sequencing libraries were uploaded into the flow-through tank and then placed in the sequencer, where the chemically modified nucleotides were combined with the DNA template strand and the DNA strand was read in both directions for bipartite sequencing. ③ Data analysis: after sequencing is completed, the instrument software automatically recognizes the nucleotides and predicts the accuracy of base detection. This assay procedure and methodology is based on a standardized commercial mNGS workflow that is widely implemented in clinical testing laboratories and commercial service providers in China.

#### 2.3.2 Anti-infective treatment

After admission, anti-infective treatment such as cephalosporin static drip and Chinese herbal medicine orally were given; 1~2 days after surgery, according to the culture results of bacterial culture and mNGS test, the sensitive antibiotic treatment of pathogenic bacteria was adjusted. In this study, eight patients underwent one-stage revision; five patients underwent two-stage revision, and two patients were treated conservatively after debridement.

### 2.4 Statistical methods

SPSS 25.0 statistical software was used for analysis. Measurement information was tested by Shapiro-Wilk test, all conformed to normal distribution, and the data were expressed as mean ± standard deviation; count information was expressed as rate, and was tested by four-cell tabular chi-square test or Fisher’s exact probability method; and the test level was α = 0.05.

## 3 Results

### 3.1 Comparison of bacterial culture method and mNGS test results

Bacterial culture method tested positive in 10 cases (66.7%) with a total of 12 pathogenic bacteria detected. Among the positive results, two pathogens were detected in one patient, three pathogens in one patient, four pathogens in one patient, and the rest were infected with a single pathogen. Single Gram-positive cocci were found in 7 cases (70.0%); Gram-positive cocci mixed with Gram-negative cocci in 1 case (10.0%); Gram-positive cocci mixed with fungi in 1 case (10.0%); and Gram-negative cocci mixed with Mycobacterium tuberculosis in 1 case (10.0%).

**Figure 1:**
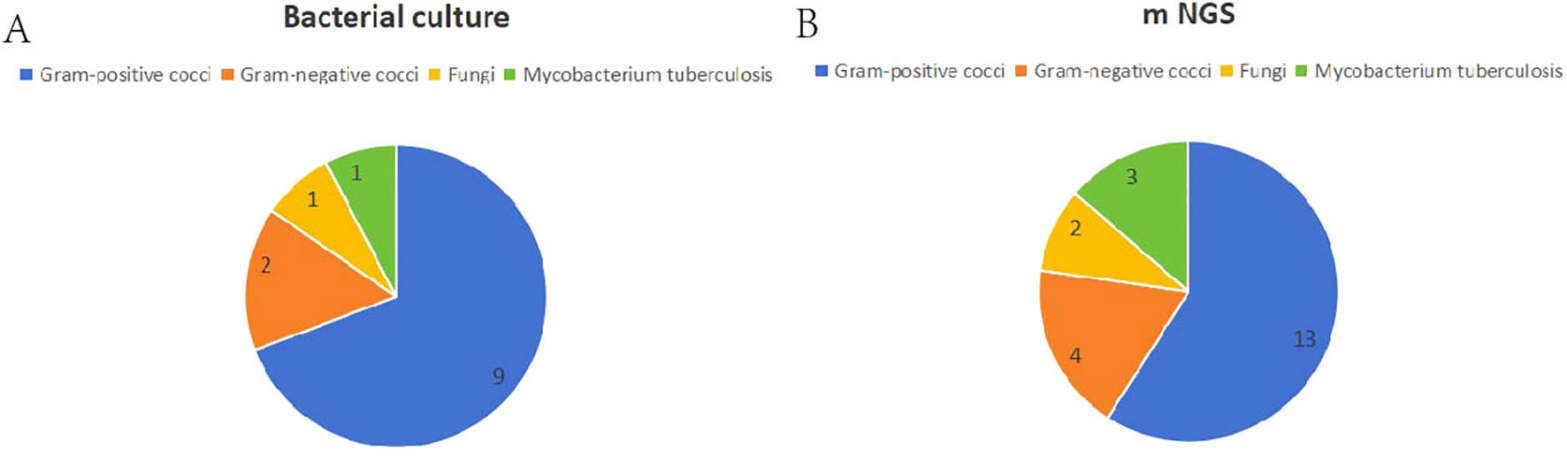
Distribution of the two detection results. **(A)** Bacterial culture results. **(B)** mNGS results.

The mNGS test was positive in 15 cases (100%), with a total of 19 pathogenic bacteria detected. Of these, 8 specimens were detected with 2 or more different bacteria, and 7 cases were infected with a single pathogen. Single Gram-positive cocci in 8 cases (53.3%); single Gram-negative cocci in 1 case (6.7%); Gram-positive cocci mixed with Gram-negative bacilli in 2 cases (13.3%); Gram-positive cocci mixed with fungi in 1 case (6.7%); Gram-positive cocci mixed with Mycobacterium tuberculosis in 1 case (6.7%); Gram-positive cocci mixed with fungi and Mycobacterium tuberculosis was detected in 1 case simultaneously (6.7%); Gram-negative cocci mixed with Mycobacterium tuberculosis in 1 case (6.7%). See **Table 3** and **Figure 1**.

**Table 3:** Comparison of bacterial culture method and mNGS detection results (cases).

Microbial species/viruses	Bacterial culture (cases)	MNGS detection (cases)
*Staphylococcus aureus*	3	4
*Staphylococcus epidermidis*	2	5
*Mycobacterium Houstonese*	1	1
*Enterobacter chuandaensis*	0	1
*Streptococcus hemolyticus*	2	3
*Mycobacterium tuberculosis*	1	2
*Desulfomonilaceae*	0	1
*Staphylococcus simulans*	1	1
*pyogenic streptococcus*	0	1
*Staphylococcus caprae*	1	1
*Enterococcus faecalis*	1	1
*Aspergillus*	0	1
*Pseudomonas aeruginosa*	1	2
*Streptococcus dysgalactiae*	0	1
*Streptococcus mitis*	1	1
*Streptococcus oralis*	1	1
*Baumannii*	1	1
Virus fragment	0	2

There was a statistically significant difference in the positive detection rates between the two methods (P < 0.05). In our group, 10 cases (66.7%) tested positive using both the bacterial culture method and mNGS test, with 5 cases showing identical pathogenic bacterial types, resulting in a 50.0% compliance rate. The time taken from sample delivery to obtaining results was (3.07 ± 0.96) days for the bacterial culture method and (1.67 ± 0.49) days for the mNGS test, with the difference being statistically significant (t=5.03, P = 0.000).

### 3.2 Antibiotic treatment

All patients were adjusted antibiotic treatment in full dosage and duration according to the test results. MNGS detected *Enterobacter chuandaensis* and *Streptococcus hemolyticus* on the basis of bacterial culture in 1 patient, and the antibiotic treatment regimen was adjusted to penicillin in combination with meropenem; in 1 patient with a negative bacterial culture, mNGS detected *Staphylococcus epidermidis* and *Mycobacterium tuberculosis*, and the antibiotic treatment regimen was changed to vancomycin in combination with rifampicin; and in 1 patient mNGS detected *Desulfomonilaceae* on the basis of bacterial culture, and antibiotic treatment was added to fluconazole; 2 patients had negative bacterial culture, mNGS detected *Staphylococcus aureus* and *Staphylococcus epidermidis* respectively, and the antibiotic treatment was changed from broad-spectrum antibiotics to rifampicin combined with levofloxacin; 2 patients’ mNGS detected *pyogenic streptococcus* and *Streptococcus dysgalactiae* on the basis of bacterial culture, and the antibiotic regimen was added to the original antibiotic regimen was changed to rifampicin combined with levofloxacin; 1 patient mNGS detected *Aspergillus* on the basis of bacterial culture, and antibiotic treatment was added with voriconazole; 1 patient with negative bacterial culture and *Pseudomonas aeruginosa* detected on the basis of mNGS, and was given ceftazidime combined with amikacin; (deleted) In 2 patients with negative bacterial cultures, viral infection was identified by mNGS analysis, and antiviral therapy was combined with valacyclovir on the basis of bacterial-sensitive antibiotics. In the remaining patients, the detection of pathogenic bacteria by the two methods differed slightly, but the antibiotic regimens were adjusted according to the test results(That is, the results of the two methods are different, but the choice of treatment regimen is the same based on the results of the test).

According to the results of bacterial culture and mNGS test culture, we took corresponding sensitive antibiotics or combined with surgery for the patients. Three days after treatment, patients’ ESR was 15–56 mm/1 h, mean 34.8 mm/1 h; CRP was 3.3-68.2 mg/L, mean 25.2 mg/L; WBC was (4.5-12.0) × 109/L, mean 7.4 × 109/L; ANC was (2.6-10.4) × 109/L, mean 5.5 × 109/L. Patients were followed up, and their follow-up period was 13 to 82 months, with a mean of 40.7 months. In 1 patient, the infection returned three months after undergoing one-stage revision surgery, and was given a stage II revision surgery of the hip joint, and was followed up for 5 years afterward, and no signs of infection were seen. There were no signs of infection in the remaining 14 cases, resulting in an infection control rate of 93.3%.

### 3.3 Typical case

#### 3.3.1 Typical case 1

**Figure 2:**
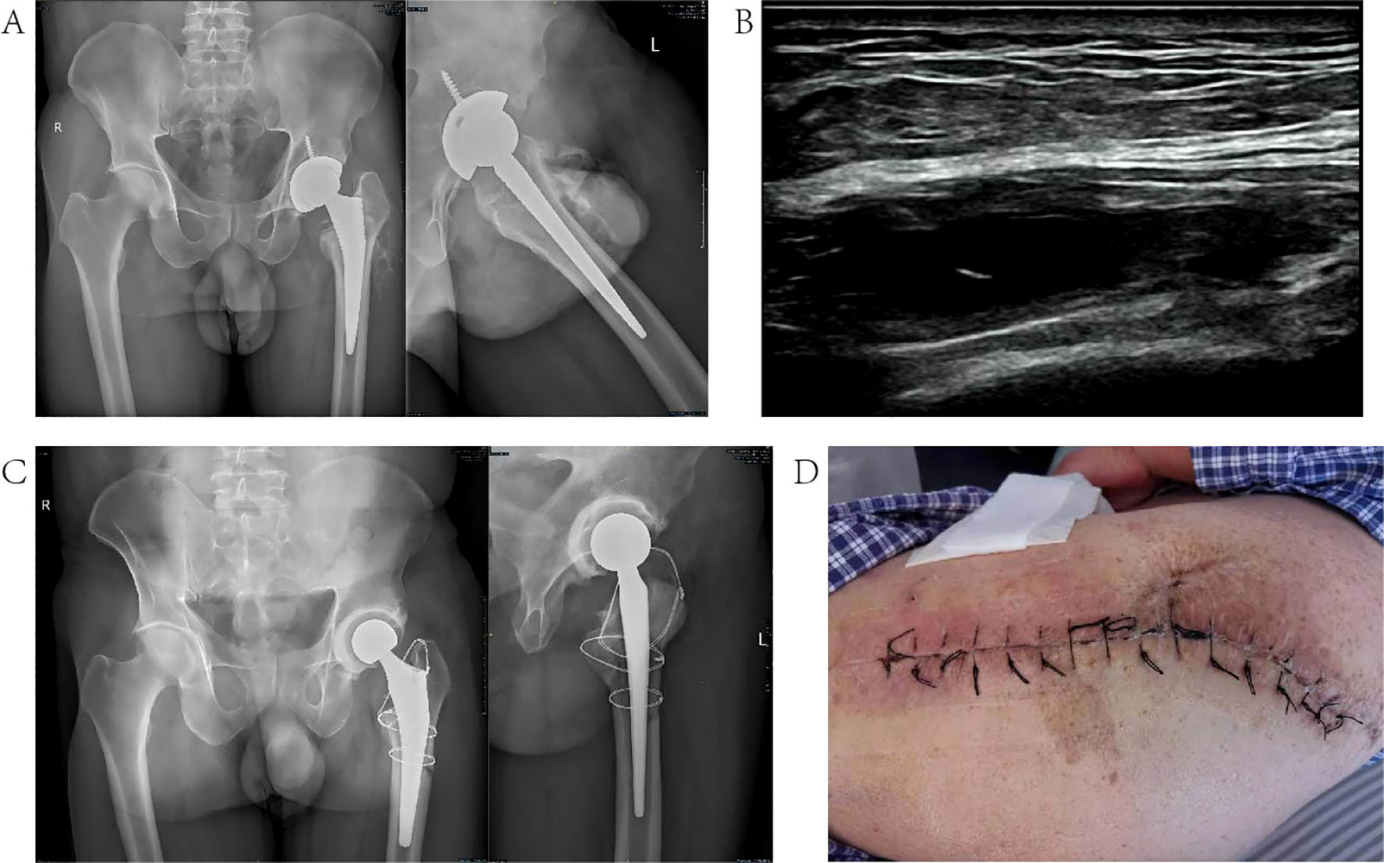
Typical case 1. **(A)** Frontal and lateral radiographs of the hip joint before the one-stage revision surgery; **(B)** localized ultrasound of the hip joint; **(C)** frontal and lateral radiographs of the hip joint after the revision surgery; **(D)** good healing of the incision without signs of infection was seen at the time of discharge from the hospital.

Patient, male, 53 years old, underwent left-sided THA for femoral head necrosis in 2019; the patient presented with redness, swelling, and pain in the left hip joint in March 2022, and was treated with local medication in a local hospital, which was ineffective; the patient came to our hospital in June 2022, and underwent a one-stage revision surgery on July 2, 2022, and antibiotic therapy was adjusted according to the results of mNGS in the postoperative period. The infection was controlled, and no signs of infection have been seen at follow-up to date. For details, see **Figure 2**.

#### 3.3.2 Typical case 2

**Figure 3:**
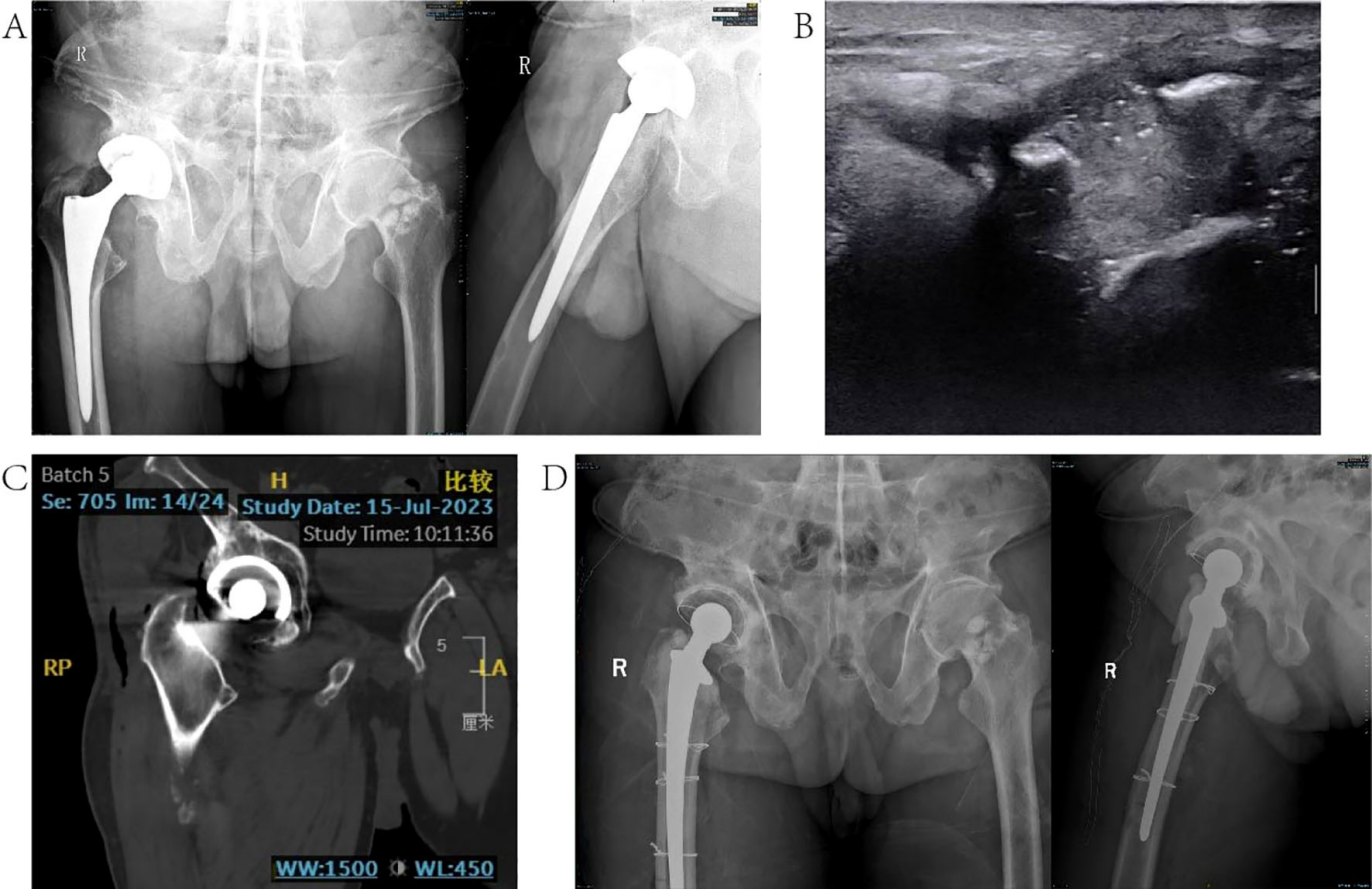
Typical case 2. **(A)** Frontal and lateral radiographs of the hip joint before the last open surgery; **(B)** localized ultrasound of the hip joint; **(C)** CT of the hip joint; **(D)** frontal and lateral radiographs of the hip joint after the open surgery.

Patient, male, 77 years old, in 2016 due to congenital hip dysplasia right THA; the patient in 2021 without obvious causes of the right hip joint incision local swelling, pain, local redness, so in the local hospital hip arthroplasty, postoperative anti-infective treatment; 2023.5 the patient’s right hip incision once again ruptured and pus, in Lianyungang People’s Hospital for In 2023.5, the patient’s right hip joint incision broke out again and pus flowed, and was treated in Lianyungang People’s Hospital. The patient came to our hospital in July 2023 and underwent right hip arthroplasty on July 22, 2022, and antibiotic treatment was adjusted according to the results of mNGS after the operation, and the infection was controlled after the operation, and no signs of infection have been seen in the follow-up so far. For details, see **Figure 3**.

## 4 Discussion

PJI is one of the most serious challenges for joint surgeons, with an incidence of 2% to 2.4% (Kurtz et al., 2012), which greatly increases patient suffering. Clinical treatments for PJI are mostly debridement, antibiotics and implant retention (DAIR), one-stage or two-stage revision (Hartzler et al., 2020; Li et al., 2020; Xu et al., 2020). A two-stage revision would be to separate debridement and replacement of the prosthesis and to revise the hip prosthesis in two operations. Relevant studies have shown that two-stage revision is significantly safer than one-stage revision (Guild et al., 2014; Frank et al., 2017; Anagnostakos and Fink, 2018), and the probability of curing PJI with two-stage revision has been reported to be more than 80% (Koo et al., 2001; Romanò et al., 2012; Guild et al., 2014).

### 4.1 Exploration of the clinical value of mNGS applied to hip PJI

As a key preoperative minimally invasive diagnostic method, synovial fluid culture has the advantage of rapidly obtaining evidence of pathogens to guide initial treatment, but its sensitivity is susceptible to antibiotic use. Prosthetic ultrasound fluid culture is the intraoperative “gold standard” for the diagnosis of PJI, which significantly improves pathogen detection by disrupting the biofilm, especially in low virulence infections and after antibiotic exposure, but must be dependent on prosthesis removal surgery. Intraoperative tissue culture is highly specific, but sensitivity is limited by sampling variability, and all three have the shared limitations of long culture cycles and insensitivity to caustic bacteria.

Microbial culture methods are recognized as the diagnostic standard for PJI, but have the disadvantage of detecting only a number of limited pathogens at a time, and suffer from problems such as inappropriate culture methods, the possibility of false positives in a single culture, and missing the optimal therapeutic window because of the long culture time, which in turn affects the outcome of the treatment (Linjie et al., 2021; Yilong et al., 2021). MNGS is a powerful tool for obtaining pathogenicity evidence, and it is a nontargeted, broad-spectrum pathogenicity screening. MNGS has excellent detection sensitivity in mixed infections or infections with negative culture of pathogenic bacteria (Wang et al., 2020; Liling et al., 2023). However, due to the complexity of PJI, the diversity of pathogens, and the complex interrelationship of microbial and host factors, the use of mNGS in the diagnosis and management of PJI is still in the exploratory stage. A study of 341 patients with PJI found that the sensitivity of NGS sequencing was 94% for detecting joint fluids of the affected limbs using two methods, while the sensitivity of routine bacterial culture was only 70% (Hantouly et al., 2023). In this study, we found that the positive rate of mNGS detection was significantly higher than that of bacterial culture method in patients with postoperative PJI after THA, indicating that mNGS can improve the detection rate of pathogenic bacteria in postoperative PJI after THA, and can not only detect pathogenic bacteria consistent with the bacterial culture method, but also detect more potential pathogenic bacteria, which can effectively avoid the occurrence of false-negative events. Once an infection occurs, its progression is usually rapid, and traditional testing methods often take 3–7 days to produce results, which is very unfavorable to early diagnosis and antibiotic application (Chang et al., 2023). In this study, the mNGS test required significantly less time than bacterial culture, indicating that the mNGS test is valuable for early clarification of pathogenic bacteria and guidance of antibiotic treatment in patients with PJI after THA.

In this study, there were 10 patients with inconsistent results of bacterial culture method and mNGS detection, of which 10 cases were detected by mNGS for more species. One patient had a history of antibiotic use within 1 month, which resulted in false-negative culture results, which might be related to the fact that the pathogenic bacteria were killed by antibiotic treatment but their DNA was still active in the short term, and the pathogenic bacteria could still be detected by mNGS. In this study, mNGS detection revealed *Staphylococcus aureus*、*Staphylococcus epidermidis*、*Streptococcus dysgalactiae*、*Pseudomonas aeruginosa*、*Enterobacter chuandaensis*、*pyogenic streptococcus*、*Streptococcus hemolyticus*、*Mycobacterium tuberculosis*、*Desulfomonilaceae*, whereas the results of the bacterial cultures were negative, which indicates that the detection rate of these types of bacteria by mNGS is higher than that of the culture method (Kurtz et al., 2018; Maimaiti et al., 2021; Maimaiti et al., 2023). Three cases mNGS detected *Staphylococcus aureus* and *Staphylococcus epidermidis* respectively, but the bacterial culture method failed to detect them, which may be related to the formation of biofilm. In this study, viral fragments were detected in 2 patients for whom we administered antiviral therapy. It is suggested that in specific cases where patients present with clinical signs highly suggestive of a viral etiology and where conventional tests are negative, detection of the corresponding viral DNA by mNGS may provide the critical evidence needed to justify targeted antiviral therapy trials.

### 4.2 Experience with mNGS-assisted treatment of PJI in hip joints

CRP and ESR are important inflammatory indicators of postoperative infection, usually patients’ CRP level can be normalized 2 weeks after surgery, if patients’ CRP level is still higher than normal 1 month after surgery, PJI is considered. Similarly, if patients’ ESR maintains a high level for 3 months consecutively after surgery, infection is considered (Fengnian et al., 2021). For superficial infections that have not yet involved the level of the joint cavity, we can adopt a conventional antibiotic regimen, i.e., vancomycin combined with cephalosporin (Zhang et al., 2020), and then adjust the use of bacterial susceptible antibiotics according to the results of tissue culture. Deep-seated infections, often manifested by biofilm-protected bacterial adherence to the prosthesis (Wildeman et al., 2020), are more drug-resistant, and conservative treatment with antibiotics alone is no longer able to control the infection, and surgical intervention is often required. If the infection does not exceed three weeks postoperatively, debridement with prosthesis preservation can be performed and antibiotics can be used in conjunction with bacterial culture results; if the infection lasts longer than three weeks postoperatively, surgical removal of the prosthesis is required. Hip revision surgery is often complex and difficult, with a high chance of postoperative complications, so thorough debridement of the joint cavity and adequate use of sensitive antibiotics are particularly important. Clinical revision of PJI of the hip mainly includes one-stage and two-stage revision. One-stage revision involves one-time removal of the infected prosthesis, placement of a new prosthesis after thorough debridement, and cemented fixation with long-acting antibiotics, combined with treatment with sensitive antibiotics for more than 6 weeks postoperatively to achieve infection control. It has been shown that one-stage revision surgery for PJI can also achieve infection cure rates comparable to those of two-stage revision surgery, as well as reduce surgical trauma, which is more conducive to functional recovery of the affected limb and improved patient satisfaction (Saul et al., 2023; Xie et al., 2024). Classical two-stage revision surgery provides satisfactory results (Kildow et al., 2022) and is usually performed in two stages, completing the removal of the infected prosthesis and the cemented absences with antibiotics, as well as the placement of a new prosthesis. In this study, eight patients underwent a one-stage revision, one patient was found to have recurrence of infection at 3-month follow-up, and a two-stage revision was performed, with eventual control of the infection; the remaining patients showed no signs of infection at later follow-up. For the choice of antibiotics, we mainly used β-lactams (amoxicillin, vancomycin, etc.), macrolides, and artificial antibiotics (quinolones, sulfonamides, etc.), relying on the high accuracy of the genetic test, and the infection was finally controlled in 14 of the 15 patients in this study, with a high cure rate of 93.3%.

### 4.3 Shortcomings and prospects

In conclusion, mNGS can rapidly and accurately find the causative organisms in patients with periprosthetic infections after total hip arthroplasty, and combined with surgery and antibiotic use, it can effectively improve the cure rate of PJI after THA. However, this study still has its limitations: (1) the number of cases included in this study is still small, and the follow-up time is short, so the results may be biased; (2) this study is a retrospective study, and the experimental design is relatively simple, and clinical studies with a larger sample size and longer follow-up time are needed to further verify the clinical efficacy; (3) Limited by the different surgical management of patients, this study was unable to collect and compare sonication and intraoperative tissue cultures in a uniform manner; (4) The broader implementation of mNGS as a routine diagnostic tool for PJI, however, faces challenges related to cost, the need for specialized laboratory infrastructure and bioinformatics support, and a longer turnaround time compared to conventional culture, which may currently restrict its use to tertiary referral centers or complex cases.

## 5 Conclusion

In conclusion, the mNGS test can improve the detection rate of pathogenic bacteria in postoperative PJI after THA, as well as greatly shorten the test time, which is important for guiding the combination of antibiotics and surgery for the treatment of patients with postoperative infections in THA.

## Data Availability

The original contributions presented in the study are included in the article/supplementary material. Further inquiries can be directed to the corresponding authors.
